# Fine-mapping reveals BcNAC153 act as a negative regulator controlling high temperature tolerance in *Brassica rapa*

**DOI:** 10.1186/s43897-025-00208-5

**Published:** 2026-04-02

**Authors:** Gaofeng Liu, Entong Li, Qian Hu, Yaliang Xu, Changwei Zhang, Ying Li, Zixin Zhang, Xilin Hou

**Affiliations:** 1 State Key Laboratory of Crop Genetics and Germplasm Enhancement/Key Laboratory of Biology and Germplasm Enhancement of Horticultural Crops in East China, Ministry of Agriculture/Engineering Research Center of Germplasm Enhancement and Utilization of Horticultural Crops, Nanjing Agricultural University, Nanjing, 210095 China; 2 Key Laboratory of Agricultural Biosafety and Green Production of Upper Yangtze River, Ministry of Education, College of Horticulture and Landscape Architecture, Southwest University, Chongqing, 400715 China; 3 Institute of Urban Agriculture, Chinese Academy of Agricultural Sciences, Chengdu, 610213 China

**Keywords:** High temperature, Non-heading Chinese cabbage, QTLs; NAC

## Abstract

High temperature stress severely reduces yield and quality in cool-climate adapted NHCC vegetable crops. It is crucial to identify high temperature response regulating genes and analyze the molecular mechanisms of NHCC in order to develop heat-resistant varieties. Here, a whole genome resequencing was conducted on NHCC001, NHCC002, and 312 F_2_ populations for QTLs mapping. A high-density bin-map containing 3608 bin markers was constructed covering 1156.88 cM, with an average adjacent interval of 0.32 cM. A major QTL for high temperature stress tolerance was identified in A01 chromosome, explaining 6.101% of phenotype variation. And BcNAC153, a NAC transcription factor, was captured and validated as a critical negative regulator for high temperature tolerance. The promoter activity of *BcNAC153* was found to be regulated by high temperature, and BcMYB44 can inhibit the transcription of *BcNAC153* by binding to the promoter of BcNAC153. In addition, this study reveals that BcCRK1 facilitates the ubiquitin-mediated degradation of BcNAC153, which affects the expression of downstream genes related to chlorophyll degradation, PCD, and ROS accumulation. Overall, this study sheds light on the molecular mechanisms underlying the response of NHCC to high temperature stress, and provides valuable insights for developing heat-resistant vegetable varieties.

**Supplementary Information:**

The online version contains supplementary material available at 10.1186/s43897-025-00208-5.

## Core

Based on QTL mapping, the negative regulatory factor BcNAC153 for heat tolerance was identified. *BcNAC153* is activated by high temperature and suppressed by BcMYB44 at the transcriptional level. It is also ubiquitinated and degraded by BcCRK1. BcNAC153 negatively affects heat tolerance by influencing chlorophyll degradation, PCD, and ROS accumulation. This discovery provides a new molecular target for enhancing stress resistance in crops.

## Genes and accession numbers

Gene information utilized in this study is is available on the BrassicaDB website of http://www.brassicadb.cn/#/. The accession number of the *BcCRK1* and *BcMYB44* genes are *Bra004600* and *Bra012149*, respectively, and the accessions of other genes are provided in Supplementary Information (Table S5-7).

## Introduction

The frequency and intensity of high temperature weather events show increase trends over the world under global warming. High temperatures can have a significant impact on the productivity and quality of crops, especially in recent decades, extreme high temperature weather events in summer have become more frequent and enhanced around the world, which can result in heavily economic losses for growers (Masson-Delmotte et al. 2021; Sun et al. 2023). To mitigate the detrimental effects of high temperature stress on crop growth and development, various measures and methods have been implemented to improve crop high temperature tolerance, including employing agronomic strategies aimed at enhancing heat tolerance, identifying and screening heat-tolerant varieties, and identifying heat-tolerant genetic loci and genes at the whole genome level for breeding (Khan et al. 2019). High temperature tolerance is an extremely complex quantitative trait, which is controlled by many genes (Kan et al. 2023).

Significant efforts have been made to map quantitative trait locus (QTLs) to enable practical marker-assisted selection (MAS) for high temperature tolerance in plants (Maloof 2003; Palanichamy 2022; Qiu et al. 2022; Zhou et al. 2022). In Arabidopsis, the heat tolerance QTL site *ER* was identified, and it was demonstrated that overexpression of the receptor-like kinase *ERECTA* gene improved the heat tolerance of rice and tomato (Shen et al. 2015). Furthermore, by conducting a genome-wide association analysis of heat tolerance in the reproductive period of 90 natural Arabidopsis varieties, a major QTL regulating heat tolerance in the reproductive period was identified. Subsequent analysis revealed that a gene cluster containing five IANs (Immune associated nucleus binding protein) genes IAN2-IAN6 within this QTL was the determining gene of this locus (Lu et al. 2021). In terms of identifying heat tolerance QTLs at the whole genome level in rice, researchers have successfully located and cloned three major thermotolerance QTL sites, TT1, TT2, and TT3 (Li et al. 2015; Yan et al. 2021; Kan et al. 2022; Zhang et al. 2022). This QTL can promptly respond to high temperature and participate in the "sanitation system," which degrades denatured proteins. This enables plant cells to remove "garbage" efficiently and enhance plant heat resistance (Li et al. 2015). Recently, another heat-resistant QTL site, *TT2*, was obtained from tropical japonica rice (Kan et al. 2022). Thus, identifying QTLs can help to explore the heat tolerance gene locus of non-heading Chinese cabbage and analyze its regulatory mechanism, facilitating the improvement of plant heat tolerance and genetic breeding.

NAC (NAM/ATAF/CUC) transcription factors (TFs) are a class of genes that encode a polypeptide with a highly conserved plant-specific N-terminal domain. Digging the function of NAC transcription factors in extremely stress-resistant plants is more helpful to reveal the mechanism of plant resistance. With the availability of complete plant genomic sequences, members of the NAC family have been identified in various species. The NAC family is one of the largest gene families in plants, with *Arabidopsis*, rice, soybean, and tobacco containing about 117, 151, 163, and 152 genes encoding NAC TFs, respectively (Singh et al. 2021). Over the past 20 years, NAC TFs have received considerable attention due to their significant roles in plant development, stress responses, and senescence (Nakashima et al. 2012; Singh et al. 2021; Han et al. 2023). Overexpressing *OsNAC045* and *OsNAC063* can enhance drought and salt tolerance in rice, and NAC transcription factor JUNGBRUNNEN1 (JUB1) enhances drought tolerance in tomato (Shahnejat-Bushehri et al. 2012). Tomato SlNAC35 regulates the expression of *ARF1*, *ARF2* and *ARF8* in the auxin signaling pathway via an ABA-dependent pathway, promoting root growth and development, enhancing drought and salt tolerance (Wang et al. 2016). In Arabidopsis, genes such as *AtNAC019*, *AtNAC055* and *AtNAC072* have been identified as key regulators of stress response and tolerance (Tran et al. 2004). A membrane-associated NAC transcription factor OsNTL3 is involved in thermotolerance in rice (Liu et al. 2020). AtNAC046 was identified as a positive regulator of chlorophyll degradation and senescence in Arabidopsis leaves, and overexpressing *AtNAC046* transgenic Arabidopsis plants were smaller than wild-type plants (Oda-Yamamizo et al. 2016). Inducible overexpression lines of *AtNAC046* in 5-day-old seedlings resulted in complete growth arrest followed by the death of the entire seedling (Huysmans et al. 2018).

As during high-temperature stress, calcium ion (Ca^2+^) becomes an important intracellular second messenger that coordinates various physiological responses in plant cell signal transduction (Dodd et al. 2010). The change in Ca^2+^ concentration activates calcium sensor proteins, which transmit the calcium signal downstream and trigger a cascade of reactions to regulate plant responses to high-temperature stress. Several major classes of Ca^2+^ sensors, including CaM and CaM-like proteins (CMLs), Ca^2+^-dependent protein kinases/CDPK-related kinases (CDPKs/CRKs), calcium/calmodulin-dependent protein kinase (CCaMK) and calcineurin B-like proteins (CBLs) (Batistič and Kudla 2012; Boudsocq and Sheen 2013). Research in maize reveals that the ZmNAC84-ZmCCaMK protein interaction coordinates ABA-triggered antioxidant responses, wherein ZmCCaMK-catalyzed phosphorylation at Ser-113 residue is indispensable for potentiating ZmNAC84's regulatory capacity in this pathway (Zhu et al. 2016). Advanced screening approaches including cDNA library construction and protein microarrays have revealed an extensive repertoire of CaM-binding transcriptional regulators (e.g., WRKY, NAC, MYB, bZIP) (Liu et al. 2024). While existing evidence has established the association between NAC transcription factors and calcium signaling pathways, the mechanistic underpinnings of NAC family members in calcium-mediated signal transduction remain insufficiently characterized. The molecular details of the dynamic interaction between the two, including how NAC senses changes in calcium ion concentration, how calcium ion signals affect the activity and function of NAC, and how NAC collaboratively regulates the expression of downstream genes with other transcription factors, all require systematic experimental verification and theoretical analysis. Further clarification of these scientific issues will provide crucial theoretical basis for elucidating the molecular mechanisms underlying the regulation of plant growth and development, as well as the response to environmental stresses.

Non-heading Chinese cabbage [NHCC, *Brassica campestris* (syn. *Brassica rapa*) ssp. chinensis] is a widely cultivated leafy vegetable with high yield and nutritional values, as it exhibits optimal growth at temperatures of 15–20°C (Li et al. 2020). High temperature is also a major abiotic stress that limits the growth and production of non-heading Chinese cabbage. Here, the QTLs affects the tolerance to high temperature (43°C) were identified. Two QTLs for the trait were identified, which were colocalized on chromosomes A01 and A07, and one of the QTL_S_ had the largest effect of 3.651% on the phenotypic variation and encompassed 89.5 Kb genomic region. A key candidate gene in this region that encodes *NAC* transcription factor, named as *BcNAC153*, was first determined for the next step of functional verification. In the following study, we found that BcNAC153 is a negative regulator for the high temperature tolerance of non-heading Chinese cabbage. The promoter activity of BcNAC153 was enhanced by high temperature, and (AT)_10_ base insertion was different in two varieties with different heat tolerance. It indicated that (AT)_10_ was a necessary sequence element for heat stress response. We also found that BcCRK1-BcNAC153 mediate the high temperature response 26S ubiquitination degrading system for heat tolerance in non-heading Chinese cabbage. In all, the discovery will provide a new target for molecular marker assisted and gene-editing breeding of high temperature tolerance in vegetable crops.

## Results

### Quantitative Trait Locus (QTL) mapping identify a candidate gene for high temperature tolerance

**Fig. 1:**
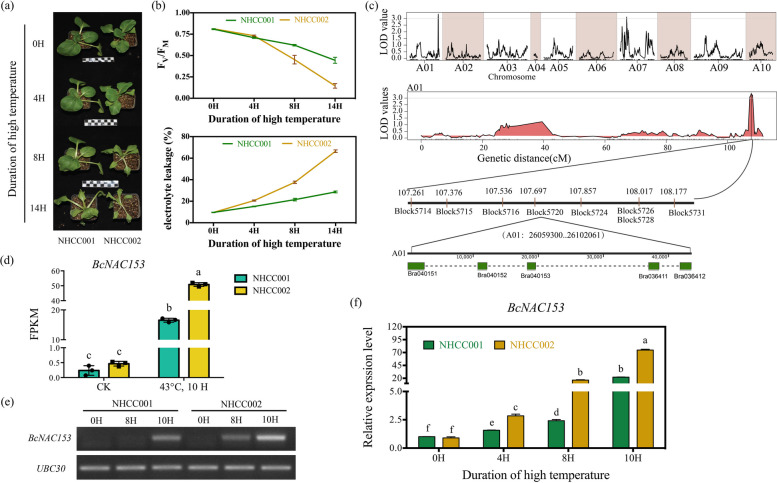
Construction of genetic map and mapping candidate genes. **a** Phenotype and (**b**) electrolyte leakage of NHCC001 and NHCC002 under normal condition (22°C) and high temperature stress (43°C). **c** Details of QTLs identifed using bins as markers. Two QTLs for HTS were identifed on chromosome A01 and A07. **d** The FPKM value in RNA-Seq database of *BcNAC153*. **e** Semi-RT-qPCR verification after 8 and 10 h (8H, 10H) 43°C treatment. CK refers controls without treatment. *UBC30* was used as the internal control. **f** The relative expression level of *BcNAC153* in NHCC001 and NHCC002 after 43°C treatment for 0, 4, 8, 10 h by RT-qPCR. Data in (**b**) and (**c**) were represent the mean ± SEM of biological triplicates. Different letters represent a significant difference at *P* < 0.05 (one-way ANOVA with Fisher’s post hoc test)

NHCC001 and NHCC002 are two elite inbred lines developed through multi-generation selfing and phenotypic selection. NHCC001, a green-stemmed line, exhibits disease resistance and heat tolerance, making it a preferred male parent in hybrid combinations. In contrast, NHCC002 is a white-stemmed self-incompatible line and is sensitive to high temperature, which serves as a predominant maternal parent in commercial F_1_ hybrid breeding programs. To identify novel genes that regulates high temperature tolerance in NHCC (non-heading Chinese cabbage), the two inbred lines with great differences in high temperature tolerance were used to construct a genetic map and evaluate high temperature tolerance. After eight hours high temperature treatment (43°C), NHCC002 exhibited severe wilting compared to NHCC001 (Fig. 1a). Consistent with the observations, F_v_/F_M_ of NHCC002 decreased obviously after four hours high temperature stress (Fig. 1b). While, the electrolyte leakage was higher in NHCC002 than NHCC001 (Fig. 1b). Accordingly, the inbred lines NHCC001 were high temperature tolerant than NHCC002. An F_2_ population comprising 312 individual plants was obtained from a cross of the two inbred lines (Fig. 1c). The F_2_ lines as well as their parents were used to construct a genetic map using. High temperature stress (HTS) treatment of 43 °C for 6H was used to assess the variation of high temperature tolerance in the F_2_ population (Fig S1a, b). F_V_/F_M_ values after HTS were used to assess the variation of high temperature tolerance in the F_2_ population which showed approximately normal distributions among F_2_ population (Fig S1b, c).The genetic map as well as the F_V_/F_M_ value were used for QTL mapping (Fig. 1c). In total, 13.46 Gbp and 13.09 Gbp clean reads were generated for the NHCC001 and NHCC002 inbred lines, which were sequenced at effective sequencing depths of about 45.97-fold and 47.26-fold, respectively (Table S1). Meanwhile, 882.56 Gb clean bases were generated for the 312 F_2_ population individuals (average 9.93-fold genome coverage), with more than 80% of the bases higher than Q30 (Table S1). For NHCC001 and NHCC002, more than 95% clean reads can be mapped to the *B. rapa* genome, and a total of 1,389,052 and 1,360,196 SNPs were identified, respectively. After filtering, a total of 686,888 SNPs were retained to determine recombination bins (bin markers). At last, A high-density bin-map with 3608 bin markers was constructed, which covered 1156.88 cM, with an average distance of 0.32 cM between adjacent bin markers (Table S2).

Quantitative trait loci (QTL) mapping was performed for high temperature tolerance using this high-density bin-map. As shown in Fig. 1d, two QTLs in A01 and A07 were identified as satisfying the LOD threshold of 3, with 6.101% and 0.49% phenotype variation explained (PVE), respectively (Table S3). We further focused on the QTL in A01 with 0.961-cM genetic distance. Eight bin markers were located in this QTL that implying the region is linked to high temperature tolerance. Among the 8 bin markers, Block5720 has max LOD values of 3.337, while the PVE was 6.101 (Fig. 1c, Table S4). This suggested that the candidate genes controlling high temperature tolerance may located within this bin marker region. Five predicted protein-encoding genes were located in Block5720 (Fig. 1c, Table S5). We further analyzed the expression patterns of these five candidate genes in NHCC001 and NHCC002 plants under high temperature stress using the transcriptome data. It was found that the *Bra040153* gene expression level was very low (FPKM < 1) under normal growth conditions in both of NHCC001 and NHCC002 (Fig. 1d). Otherwise, *Bra040153,* named as *BcNAC153* in the following, were strongly heat induced in different degree in NHCC001 and NHCC002 under 10-h (10 H) high temperature stress (Fig. 1d). *Bra040153* encodes nascent polypeptide-associated complex protein (NAC), which were homology to *AtNAC046* (*AT3G04060*) in Arabidopsis. Semi-qRT-PCR and qRT-PCR results showed that the relative expression level of *BcNAC153* in NHCC002 was significantly higher than that in NHCC001 after 43°C for 8 h (Fig. 1e and f). These results clearly indicated that *BcNAC153* responded to high temperature stress and showed different expression levels during the stress at NHCC001 and NHCC002.

### The expression of *BcNAC153 gene* was high temperature induced and can be repressed by transcription factor BcMYB44

To further prove the function of *BcNAC153* on high temperature stress, the transgenic Arabidopsis strain with overexpression of *BcNAC153* gene was constructed. Phenotypic observation showed that the *BcNAC153* overexpression Arabidopsis plants were much smaller than the wild type Col-0 plants (Fig. S2). The high temperature tolerance of transgenic lines was further identified. After being treated at 43°C for 8 h (8H), the leaves of the overexpression lines were more wilted than those of the wild type Col-0 (Fig. S2). Consistent results were also obtained in the experiment of transient transformation of *BcNAC153* in non-heading Chinese cabbage (Fig. S3). Overexpression of *BcNAC153* lines exhibited significantly reduced heat resistance in the leaves (Fig. S3a, b), with a higher electrical conductivity (Fig. S3c). This result preliminarily indicates that overexpression of *BcNAC153* leads to the decrease of high temperature tolerance of NHCC.

**Fig. 2:**
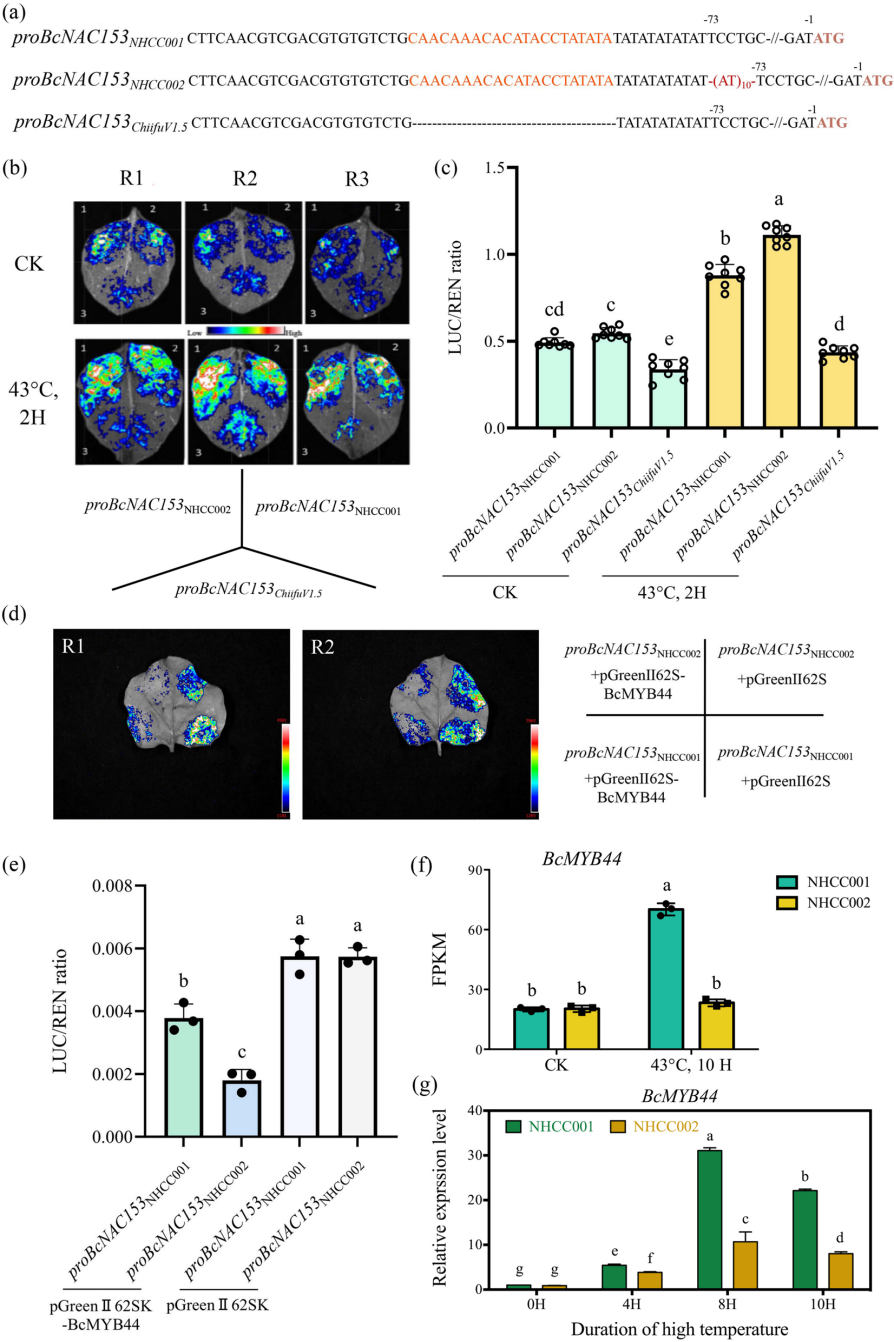
*BcNAC153* was high temperature induced and repressed by BcMYB44 (**a**) Schematic diagram indicating the variants of *BcNAC153* promoter in NHCC001 and NHCC002 and its mutated version. **b** Luminous intensity taken by CCD freezing camera. **c** Promoter activity analysis in tobacco leaves before and after treat with 43°C for 2 h. **d** Luminous intensity taken by CCD freezing camera. **e** BcMYB44 binds to *proBcNAC153* to inhibit its expression. LUC/REN is the average ratio of the bioluminescence of firefly luciferase to that of Renilla luciferase. **f** The FPKM value in RNA-Seq database of *BcMYB44*. **g** The relative expression level of *BcMYB44* in NHCC001 and NHCC002 after 43°C treatment for 0, 4, 8, 10 h by RT-qPCR. Data in (**f**) and (**g**) were represent the mean ± SEM of biological triplicates. Different letters represent a significant difference at *P* < 0.05 (one-way ANOVA with Fisher’s post hoc test)

We further cloned and sequenced the promoter sequence of *BcNAC153* from NHCC001 and NHCCC002 respectively. Sequence alignment results showed that there was a variation in the number of (AT)_n_ insertions upstream of the starting codon ATG, and *pro*NHCC002 had more (AT)_10_ base insertions than *pro*NHCC001 by comparing to the reference genome (Fig. 2a). To determine the effect of different base insertions on promoter activity, a segment of sequence upstream of AT was deleted and mutated, and promoter activity was analyzed in tobacco (Fig. 2b and c). The result showed that promoter of NHCC001 and NHCC002 activities were significantly enhanced after high temperature treatment, indicating that BcNAC153 promoter activity was regulated by heat stress. At the same time, the promoter activity of the deleted mutant promoter sequence is still weak after heat treatment, so the missing promoter sequence is a necessary sequence element for heat stress response. Selected varieties with different heat resistance were used to detect the mutation of (AT)_n_ base insertion in the promoter region (Fig. S3). The result showed that heat tolerance varieties presented with less number of (AT)_n_ insertion (6–8), while the heat sensitive varieties contained much more (AT)_n_ insertion (18–26) (Fig. S4). It is convinced that (AT)_n_ base insertion contributed to the promoter activity under high temperature.

To investigate the molecular mechanism of BcNAC153-induced heat tolerance, *proBcNAC153*_*NHCC002*_ were used as baits to screen yeast one-hybrid libraries. A cDNA fragment showing homology to BcMYB44 was identified to bind to *proBcNAC153* (Table S6). As the Arabidopsis homologous gene *AtMYB44* has been reported to play vital roles in responses to abiotic stress, we hypothesize that BcMYB44 is a potential regulator (Jung et al. 2008; Masrur et al. 2013; Wang et al. 2021). Using the Dual-Luc system in tobacco leaves, we confirmed that BcMYB44 could repress the activity of the BcNAC153 promoter in both NHCC001 and NHCC002, however, BcMYB44 had a more significant inhibitory effect on *proBcNAC153*_NHCC002_ (Fig. 2d, e). The expression level of *BcMYB44* in NHCC001 was significantly higher than that in NHCC002 after 43°C for 10 h (Fig. 2f), and qRT-PCR results showed the same tendency (Fig. 2g), which just in contrast to *BcNAC153* (Fig. 1d, f).

### BcCRK1 interacting with BcNAC153 and promoting BcNAC153 degradation

**Fig. 3:**
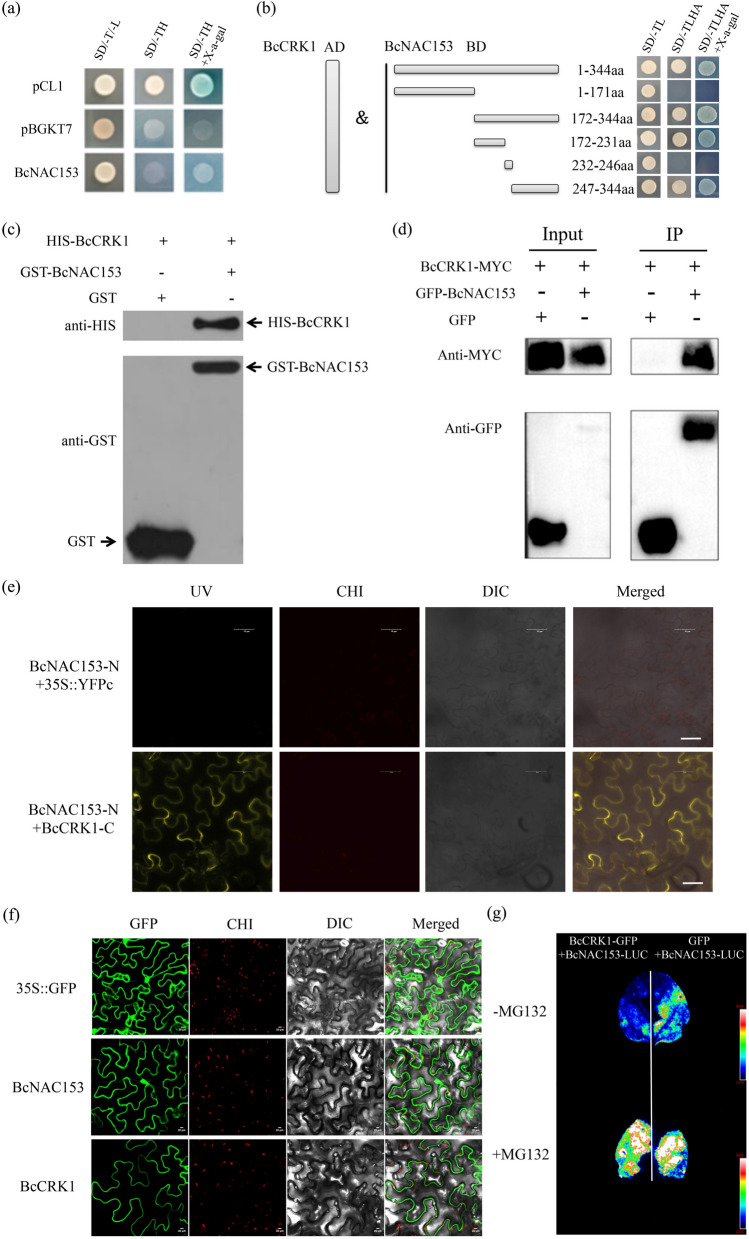
Interaction between BcNAC153 and BcCRK1. **a** Yeast hybrid system analysis of the transcriptional autoactivation activity of BcNAC153. Yeast cells harboring pBGKT7-BcNAC153 and empty vector control (pGBKT7, BD) were unable to grow on SD/-Trp-His (SD/-TH) medium, and also did not turn blue on SD/-Trp-His with X-α-gal-coated medium. Meanwhile, cells carrying pCL1, which act as a positive control, grew well on both media. **b** Interactions were tested by yeast two-hybrid screening. The yeast was transformed using an empty vector, a fusion of BcNAC153 (Full length amino acid sequence, 1–344 aa), BcNAC153 (1–171 aa, 172–344 aa, 172–231 aa, 232–246 aa, 247–344 aa) to the Gal4-binding domain (BD), or a fusion of BcCRK1 to the Gal4 activation domain (pGADT7, AD). Yeast growth on nonselective (-LT) and selective (-LTAH without or with X-α-gal) SD medium is shown. (The sequence segmentation information of protein fragments is shown in Fig. S4). **c** Pull-down assay. GST-BcNAC153 was pulled-down by HIS-tagged BcCRK1, and HIS-BcCRK1 was also pulled-down by GST-tagged BcNAC153. **d** Co-IP assay. GFP-BcNAC153 was dragged by immunoprecipitation of MYC-tagged BcCRK1, and BcCRK1-MYC was dragged by immunoprecipitation of GFP-tagged BcNAC153 N. benthamiana leaves were agroinfiltrated with BcCRK1-MYC and GFP-BcNAC153. Two days after agroinfiltration, total protein extracts were immunoprecipitated with an anti-FLAG antibody. GFP-BcNAC153 was detected in these fractions with an anti-GFP antibody, and BcCRK1-MYC was detected in these fractions with an anti-MYC antibody. **e** BiFC assay. BcNAC153 was fused to the N-terminal fragment of YFP, while BcCRK1 was fused to C-terminal fragment of YFP. The interaction between BcNAC153-N and 35S::YFPc was used as negative controls. Representative images captured after 72 h infiltration. **f** The subcellular locazation of GFP activity in cells tobacco leaves. A transient transform of 35S::GFP-BcNAC153 and 35S::GFP-BcCRK1 fusion constructs expressed in cells were detected by CCD camera, respectively. GFP: green fluorescence channel; CHI: chloroplast fluorescence channel; DIC: images captured under bright light; Merged: merged images of the above. Bar = 30 μm. **g** The degradation of BcNAC153 protein by BcCRK1 in *N. benthamiana* leaves. MG132 act as a proteasome inhibitor that can prevents the degradation

To elucidate the molecular mechanism modulated by BcNAC153 in the high temperature signaling pathway, we performed yeast two-hybrid screen against NHCC stress cDNA library using BcNAC153 as the bite protein on the fact that the nonexistence of transcriptional self-activation activity (Table S7, Fig. 3a). A cDNA fragment showing homology to BcCRK1 was identified to interact with BcNAC046 in yeast cells. The point-to-point verification in yeast cells again proved that BcCRK1 could interact with BcNAC153 in yeast cells (Fig. 3b). BcNAC153 was further cloned into pGBKT7 vector, and its interaction fragments were identified. The results showed that the 172–231 amino acids and 247–344 amino acid fragments of BcNAC153 protein were the interacting sequences with BcCRK1 (Fig. 3b). Therefore, BcCRK1 was hypothesized as a potential interaction protein with BcNAC153 for high temperature stress response. Pull-down (Fig. 3c), Co-IP (Fig. 3d) and bimolecular fluorescence complementation (BiFC) (Fig. 3e) assays also confirmed the interaction between BcCRK1 and BcNAC153. The BiFC in tobacco leaves proves that the interaction occurs on the cell membrane (Fig. 3e). The localization detection indicates that BcNAC153 was localized in the nucleus and membrane, and BcCRK1 was in the membrane of cells (Fig. 3f). To verify how BcCRK1 regulates BcNAC153 at the protein level, BcNAC153-LUC was expressed together with BcCRK1 in tobacco leaves. MG132 was used as a proteasome inhibitor that can prevents the protein degradation. As shown in Fig. 3g, the luciferase signal was significantly decreased without supplement of MG132 compared to the right control. The phenotype can be effectively blocked by infiltrating the proteasome inhibitor MG132. This indicates that BcCRK1 might facilitate the ubiquitin-mediated degradation of BcNAC153.

### *BcNAC153* decreased the high temperature tolerance by downstream genes related to chlorophyll degradation, programmed cell death and ROS accumulation.

**Fig. 4:**
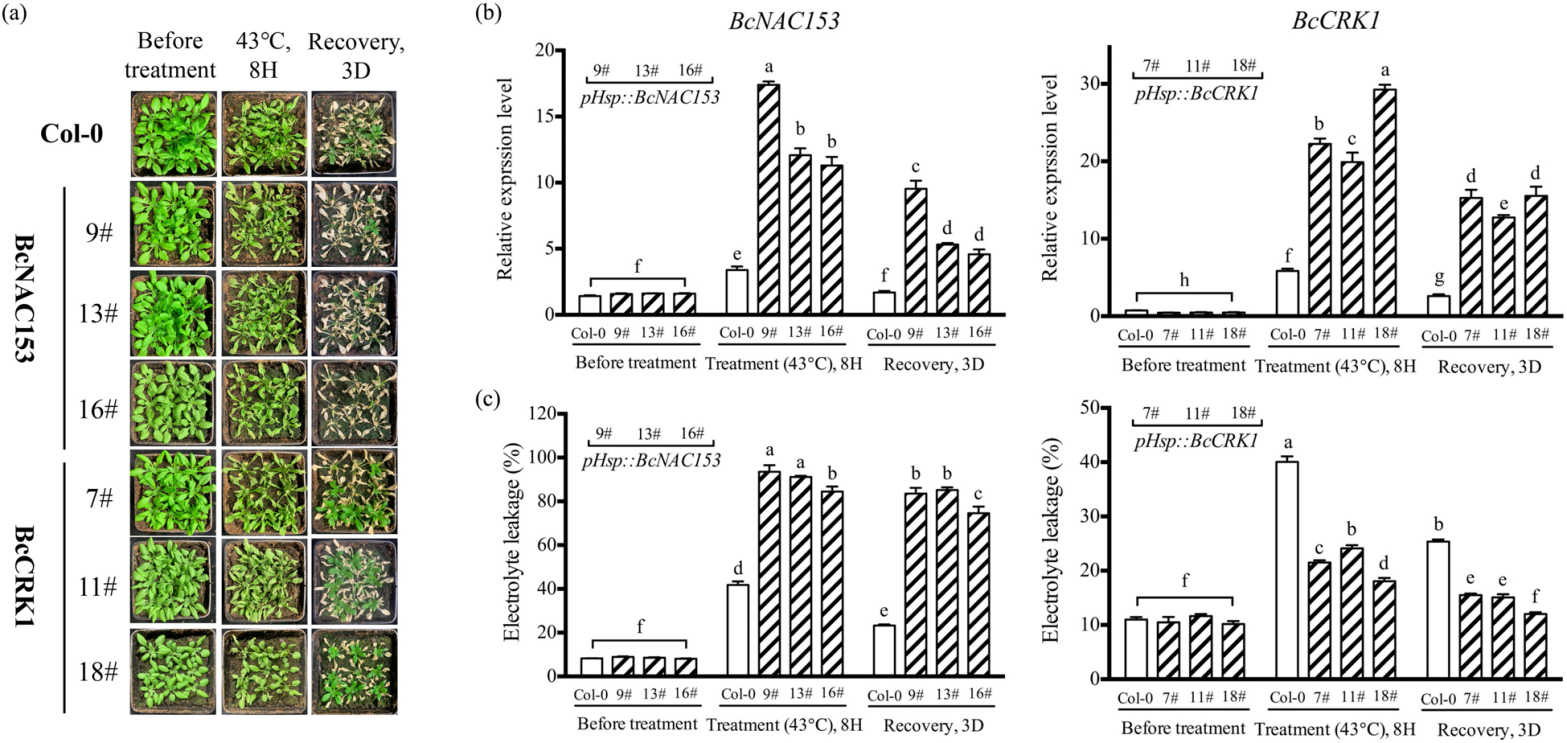
The genetic analysis of *BcNAC153* and *BcCRK1* on the high temperature tolerance. **a** Phenotype of *pHSP::BcNAC153* and *pHSP::BcCRK1* transgenic plants before and after 43 °C treatment, as well as recovery for 3 days on normal conditions. **b** Transcript levels and (**c**) Electrolyte leakage of *BcNAC153* and *BcCRK1* in wild-type Col-0 and transgenic plants before and after 43 °C treatment, as well as recovery for 3 days on normal conditions. Data in (**b**) and (**c**) were represent the mean ± SEM of biological triplicates. Different letters represent a significant difference at *P* < 0.05 (one-way ANOVA with Fisher’s post hoc test)

**Fig. 5:**
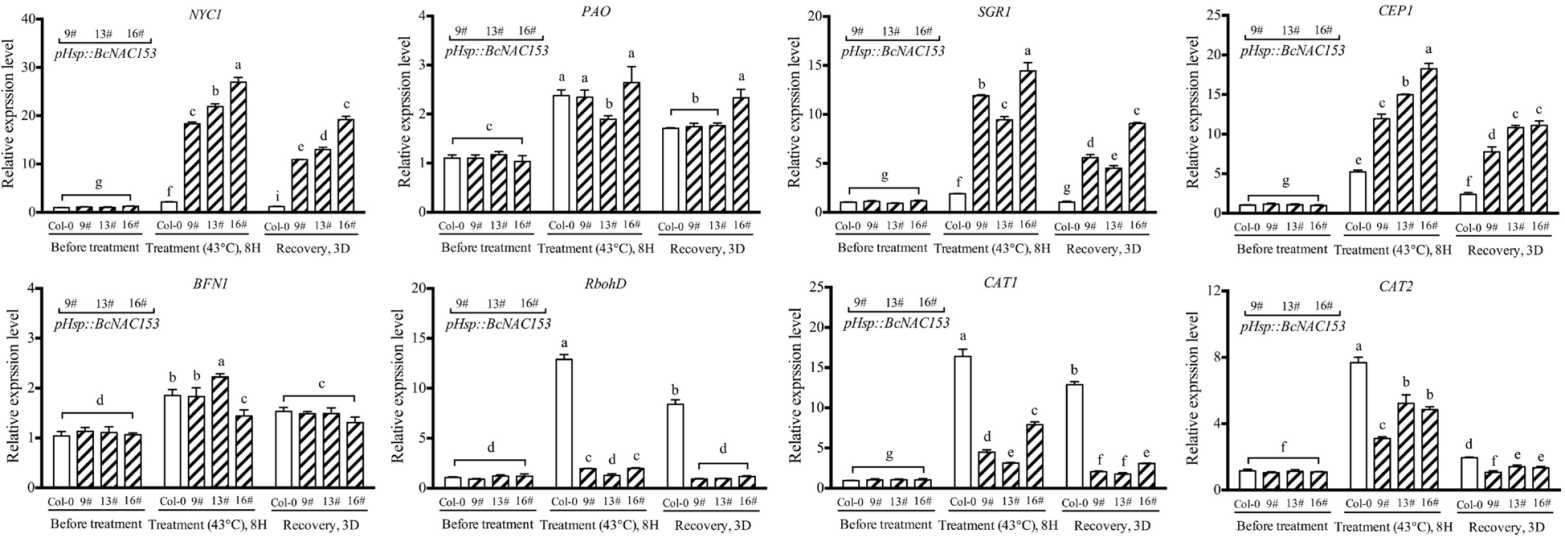
Transcript levels of the target downstream senescence-associated genes in wild-type Col-0 and *pHsp::BcNAC153* transgenic plants before and after 43 °C treatment, as well as recovery for 3 days on normal conditions. Data was represent the mean ± SEM of biological triplicates. Different letters represent a significant difference at *P* < 0.05 (one-way ANOVA with Fisher’s post hoc test)

As the overexpression transgenic lines induced by *35S* promoter grew abnormally, which were smaller than the wild-type Col-0 plants. We reconstructed the fusion plasmid of heat shock-induced promoter *pHsp*. In the three transgenic lines of *BcNAC153*, the plant showed severe wilting compared with the wild type Col-0. The expression of *BcNAC153* was actually activated after 8 h of 43 °C treatment (Fig. 4c). The percent of electrolyte leakage was significantly increased, and after 3 days recovery, there was no significant improvement (Fig. 4c). In contrast, in *BcCRK1* transgenic lines, when the expression of *BcCRK1* was activated by heat induction, the percent of electrolyte leakage was lower than that of Col-0 (Fig. 4c). After 3 days of recovery, they basically returned to normal (Fig. 4c). These results suggest that *BcNAC153* is a negative regulator of high temperature tolerance, and may mediated by BcCRK1. Moreover, a set of downstream senescence-associated genes in inducible overexpression of *BcNAC153* Arabidopsis were quantified. The result shown that the genes participating in the regulation of chlorophyll degradation (*NYC1*, *PAO1*, *SGR1*), programmed cell death (*CEP1*, *BFN1*), were significantly induced. While, The genes related to reactive oxygen species accumulation (*RbohD*, *CAT1*, *CAT2*), were significantly down-regulated (Fig. 5).

## Discussion

High temperature stress has emerged as a critical constraint for vegetable production during the summer, and autumn seasons. Elucidating the genetic mechanisms underlying heat tolerance is fundamental for breeding new varieties with improved heat resilience (Wahid et al. 2007; Ohama et al. 2017; Sliddiqui et al. 2017). High temperature tolerance breeding assumes paramount importance in non-heading Chinese cabbage, given that heat tolerance levels vary across different varieties. In the preliminary stage of this study, we re-sequenced the genomes of two parental materials, NHCC001 and NHCC002, displaying differential levels of heat tolerance (Fig. 1a, b). Additionally, we constructed an F_2_ isolation population and developed a high-density genetic linkage map for non-heading Chinese cabbage (Fig. 1c, Fig. S1a). To map QTLs for high temperature stress, F_V_/F_M_ was applied as a phenotypic index and successfully identified two heat tolerance-related QTLs (Fig. 1c, Fig. S1b, c). Finally, *BcNAC153*, encoding an NAC transcription factor was identified as a key candidate gene. While the homologous genes in Arabidopsis have been reported to induce chlorophyll degradation and leaf senescence (Mahmood et al. 2016; Oda-Yamamizo et al. 2016; Huysmans et al. 2018; Chen et al. 2022), its molecular mechanism in response to high temperature stress signals remains elusive in non-heading Chinese cabbage. The present study seeks to investigate the molecular mechanism of BcNAC153 in high temperature tolerance to identify genetic functions and elucidate the heat stress response. The study outcomes will provide a theoretical framework for molecular breeding of heat-tolerant non-heading Chinese cabbage, offering profound scientific and practical implications.

In light of the negative effects of *BcNAC153* in response to high temperature stress, its expression levels and timing were found to differ between NHCC001 and NHCC002 cultivars as shown in Fig. 1c-f. The activities of both *proBcNAC153*_*NHCC001*_ and *proBcNAC153*_*NHCC002*_ were significantly enhanced by high temperature treatment, indicating that BcNAC153 promoter activity is regulated by heat stress, and the weak promoter activity of the missing mutant promoter sequence after high temperature treatment (Fig. 2a-c) suggests that this missed sequence is a crucial element for the high temperature response. Promoter activity analysis revealed that *proBcNAC153*_*NHCC002*_ exhibited more (AT)_10_ base insertions than *proBcNAC153*_*NHCC001*_ (Fig. 2b, c). As it has been verified in 8 different inbred lines, the amount of AT insertion can be used as a correlation index to evaluate the heat tolerance of non-heading Chinese cabbage (Fig. S4). These findings may have implications for the future screening of high temperature resistant NHCC varieties.

Futher, a transcription factor BcMYB44, which obtained from the screening of the yeast library, may be related to the transcription regulation of *BcNAC153* (Table S6). BcMYB44 has been demonstrated to promote leaf anthocyanin synthesis and enhance plant drought tolerance in NHCC (Hao et al. 2022). BcMYB44 is homologous to AtMYB44 in Arabidopsis, and the *AtMYB44* overexpression lines present with resistance to abiotic stress (Seo et al. 2012; Wang et al. 2024). In this study, BcMYB44 was found to bind to the promoter sequence of BcNAC153, thereby inhibiting its transcription (Fig. 2d, e). Upon the occurrence of high temperature, the expression of *BcMYB44* and *BcNAC153* exhibit an inverse trend (Fig. 2d, e). The findings suggest that BcMYB44 can regulate the expression of *BcNAC153*, thereby influencing the plants' heat tolerance. This study has also expanded the functional understanding of BcMYB44 in NHCC, and future research can further explore the heat resistance capabilities of BcMYB44.

The regulatory network of NAC transcription factors is complex and involves interactions with multiple factors (Nakashima et al. 2012; Singh et al. 2021). Among these factors of non-heading Chinese cabbage, BcCRK1 was identified as an interaction partner of BcNAC153 through yeast two-hybrid screening, warranting further investigation. CRKs are a class of protein kinases that play critical roles in plant signal transduction pathways (Abu et al. 2018; Delormel and Boudsocq 2019). Previous studies have demonstrated that Arabidopsis AtCRK1 can phosphorylate the heat shock transcription factor AtHSFA1a, thereby promoting the heat shock response (Liu et al. 2008). It was found that AtCRK3 can regulate its ubiquitination of FLS2 and therefore affect plant immune response (Lu et al. 2011). Additionally, AtCRK3 has been shown to interact with cytosolic glutamine synthetase AtGLN1.1, suggesting its involvement in leaf senescence (Li et al. 2006). However, the specific phosphorylation substrates (interacting proteins) and phosphorylation modification sites of CRKs remain largely unknown (Delormel and Boudsocq 2019). To investigate the regulation of BcNAC153 by BcCRK1, we performed an assay of BcNAC153 protein degradation in *N. benthamiana* leaves treated with MG132, which revealed that BcCRK1 can modulate BcNAC153 stability through the 26S proteasome pathway, thereby contributing to plant heat resistance response (Figs. 3g and 4). Furthermore, the analysis of interaction sites predicted the presence of a phosphorylation site and a ubiquitination site between the two amino acid sequences of BcNAC153 (Fig. S5). Based on these findings, we speculate that the ubiquitination degradation of BcNAC153 by BcCRK1 in non-heading Chinese cabbage also involves BcNAC153 phosphorylation modification, which warrants further investigation.

Quantitative analysis demonstrated up/down-regulated expression of a set of senescence-associated genes in plants with inducible overexpression of *BcNAC153*, specifically *NYC1*, *PAO*, *SGR1*, *CEP1*, *BFN1*, *RbohD*, *CAT1*, and *CAT2*, which are known to participate in the regulation of chlorophyll degradation, programmed cell death (PCD), and reactive oxygen species (ROS) accumulation (Fig. 5). Notably, AtNAC046, a homolog of BcNAC153 in Arabidopsis, has been reported to bind directly to the promoter regions of chlorophyll catabolic genes, such as *NYC1*, *SGR1/2* and *PAO*, involved in chlorophyll degradation and PCD (Oda-Yamamizo et al. 2016). Additionally, previous study indicates that ANAC087, in conjunction with ANAC046, was identified as regulators of developmental PCD in the columella root cap (Huysmans et al. 2018). AtORE1 (ANAC092) positively modulate leaf senescence by regulating the expression of *BFN1* (Matallana-Ramirez et al. 2013). In Arabidopsis, overexpression of either/both *CEP1* or/and *βVPE* proteases partially recover pollen vitality in the *myb2* background. *MYB2* is involved in tapetal PCD and pollen development by directly regulating the expression of the protease *CEP1* and *βVPE* and establishes a transcription factor/proteases regulatory and activated cascade (Guo et al. 2022). Here, we reported BcNAC046, may also function as an important hub linking PCD, ROS, chlorophyll degradation, and high temperature tolerance. In summary, our results not only uncover a novel TF of NAC mediating high temperature response, but also deepen the understanding of the complex regulatory network in high temperature tolerance. The further studies will focus on regulatory mechanism the homeostasis of BcNAC153 protein, as well as identify efficient downstream direct executors for BcNAC153 during high temperature stress, using transgenic non-heading Chinese cabbage and mature molecular verification methods. By doing so, we hope to elucidate a novel pathway for regulating heat tolerance and identify new targets for enhancing the heat tolerance of vegetable crops.

**Fig. 6:**
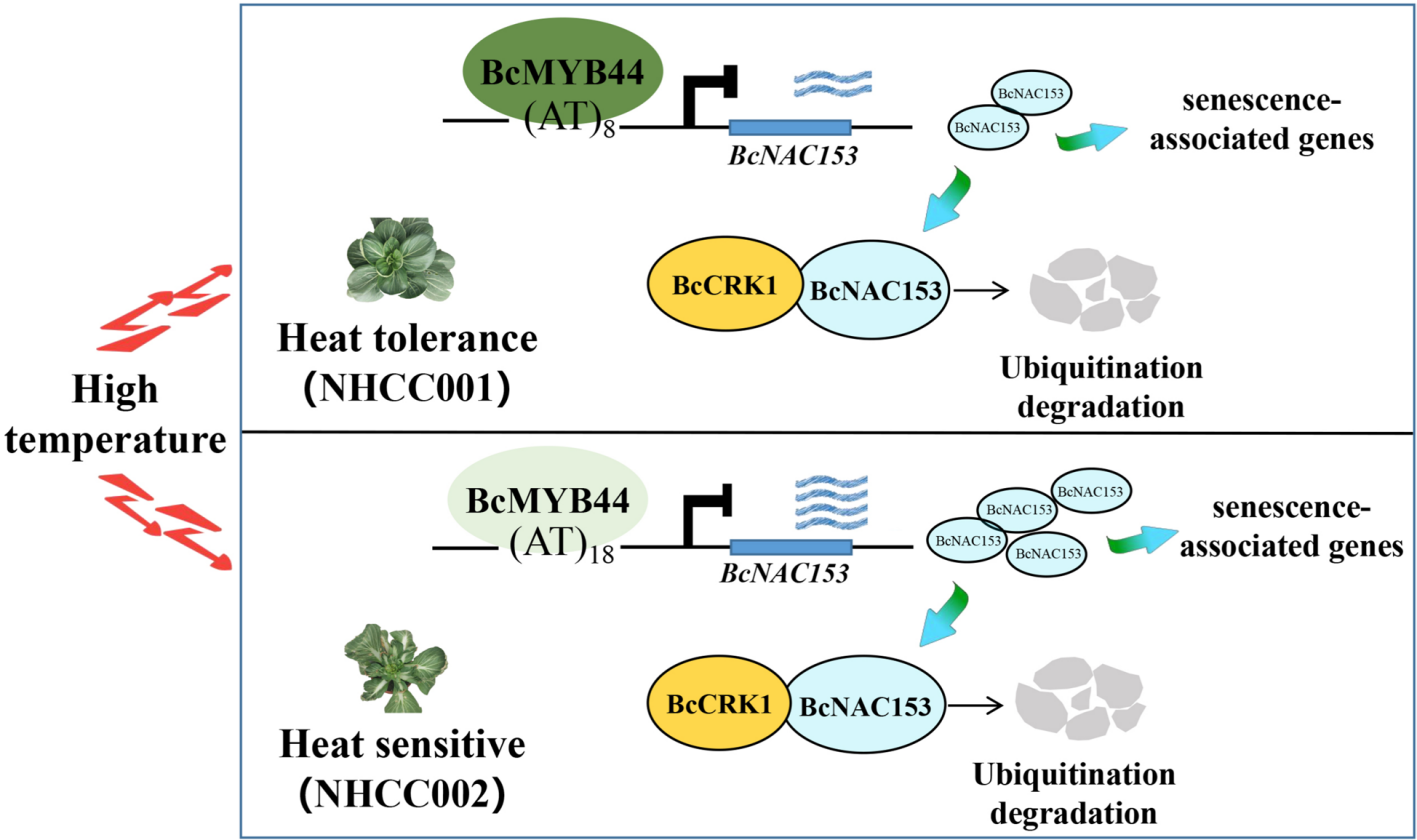
Schematic representation of BcNAC153 and BcCRK1 mediating the integration of high temperature tolerance. *BcNAC153* was highly expressed in heat sensitive cultivar NHCC002 and low in heat resistant cultivar NHCC001, while *BcMYB44* was the opposite. BcMYB44 directly binds to the promoter of BcNAC153 to inhibit its expression. BcCRK1 is activated under the high temperature, and can facilitate the degradation of BcNAC153 through 26S proteasome pathway, thus removing the combination with the down-stream gene, leading to the high heat resistance. The ubiquitination degradation of BcNAC153 may require BcCRK1 to phosphorylate it first, which may involve ubiquitination after phosphorylation modification. Quantitative validation preliminarily revealed the target downstream senescence-associated genes that may be affected directly or indirectly

The production of non-heading Chinese cabbage during summer or after exposure to high temperatures is often characterized by slow growth, high rates of seedling death, bitterness, a significant increase in fiber content, and leaf yellowing. As such, breeding heat-resistant varieties has become a critical objective for developing new non-heading Chinese cabbage cultivars. The identification of heat-resistant genes and the analysis of molecular mechanisms (Fig. 6) will provide a foundation for breeding new varieties with increased heat resistance. Additionally, it is important to continue building and improving the heat tolerance regulation network of non-heading Chinese cabbage and further link the metabolic regulation network, as this will pave the way for more in-depth research in the future. Moreover, the expression patterns and regulatory networks of these genes can be applied to other species, and the research model of this regulatory network can also be extended to other environmental factors.

## Materials and Methods

### Plant materials and growth conditions

SZQ (NHCC001) and AJH (NHCC002) were used as parents to construct the F_2_ population in this study, which were developed and stored at Nanjing agricultural university. The two lines were also used as the parental materials for the elite hybrid cultivars, which is high temperature tolerant variety and widely cultivated in China. 312 F_2_ offspring were obtained from NHCC001 (♂) × NHCC002 (♀). The seeds of non-heading Chinese cabbage were germinated in petri dish on wet filter paper. After germination, they were transferred to plug for normal soil-growth on vermiculite in a greenhouse with a constant temperature of 23 °C, a 16 h light/8 h dark photoperiod cycle, and 60% relative humidity. Four-leaf stage seedlings were transferred to another setting to 43 °C without light in advance for heat treatment. All Arabidopsis plants for phenotype observations and statistics were soil-grown under a constant temperature of 22 ± 2 °C, a 16 h photoperiod, and 70% relative humidity in addition to special treatments. Two weeks later, the seedlings were moved to 43 °C growth room without light for 8 h high temperature treatment, and recovery for 3 days.

### Determination of FV/FM and electrolyte leakage

Chlorophyll fluorescence F_V_/F_M_ was used to examine variation of high temperature tolerance among the progeny using PAM-2000. The final F_V_/F_M_ of plants were obtained from the average of three leaves F_V_/F_M_. Leaves were cut into small pieces (around 5 mm), and incubation for 2 h in 50 mL centrifuge tube with 20 mL ddH_2_O. Measure the electrical conductivity of the solution by a conductivity meter. Boil the samples for 15 min, then measure the conductivity of the boiled samples and record it as the total electrolyte leakage. Divide the initial conductivity (measured after incubation) by the total conductivity (measured after boiling), and multiply the result by 100, which achieved the percentage electrolyte leakage.

### Bin map construction and QTL analysis

Total genomic DNA was extracted from each parental and F_2_ population leaf sample following the manufacturer’s protocols with the Plant Genomic DNA Kit (TIANGEN, Beijing, China). Paired end sequencing libraries were sequenced using an Illumina HiSeq X Ten platform (Illumina, San Diego, CA, USA). The Burrows Wheeler Aligner (BWA) was used to align the clean reads from each sample against the reference genome (Li 2009). QTL analysis and mapping were initially carried out by composite interval mapping (CIM) of the rQTL package to identify genomic regions responsible for high temperature tolerance (Broman et al. 2003). The significance thresholds were determined using 1,000 permutations, in which the threshold value was set as 3.0. All phenotyping data were used for QTL analysis.

### RNA extraction and quantitative analysis

Total RNA was extracted, using the TRIzol reagent (Invitrogen, Carlsbad, CA, USA), as recommended by the manufacturer. A 1 μg aliquot of RNA was used for the synthesis of the cDNA first strand, using a PrimeScriptTMRT reagent Kit containing gDNA Eraser (Takara, Shiga, Japan). The cDNA was used as the template for qRT-PCRs based on Fast SYBR Green Master Mix (www.bimake.com). The relative transcript abundances were calculated using the 2 − ΔΔCT method (Livak and Schmittgen 2001). Semi-qRT-PCR test is quantitative by agarose electrophoresis. PCR primer pair in Table S8 for amplification.

### Construction and screening of the yeast library

The construction and screening of the yeast library was handled by Yuanbao Biotech (Nanjing, China). SD/-His-Leu-Trp dropout 3-AT culture screening plates and the Y187 yeast strain were used to screen the yeast library. High-throughput sequencing was performed after colony collection. The raw data was transferred to fasta using fq2fa, the paraments was outfmt 6, e value was 1e^−4^.

### Promoter activity detection and transient dual-luciferase reporter assay

The fragment of the *BcNAC153*_*NHCC001*_, *BcNAC153*_*NHCC002*_ and *BcNAC153*_*chiifuV1.5*_ promoter was respectively cloned and inserted into the *pGreenII62S* vector. The detailed methods and procedures refers to the published literatures (Zhang et al. 2023a, b). *A. tumefaciens* strain GV3101 harbouring targeted fragments was grown in infiltration medium to an OD600 of 0.5 and then introduced into the leaves of the ~ 4-week-old *Nicotiana benthamiana* plant. A CCD camera was used to observe luciferase activity after 48–96 h. The Dual-Luciferase Reporter Assay System E1960 (Promega, Madison, cat. #e1910) was used to measure the fluorescence intensity of luciferase and renilla (REN). The relative LUC/REN ratios were used to represent the activity of the promoters.

### Yeast two-hybrid assay

The coding regions of *BcCRK1* was amplified and cloned into pGADT7 (Clontech), and co-transformed with BcNAC153-pGBKT7 into yeast Y2H Gold strain. The detailed methods and procedures refers to the published literatures (Zhang et al. 2023a, b). pGBK-53 and pGADT were used as positive controls, while pGBK-Lam and pGADT were used as negative controls. The yeast cells were grown on -Leu/-Trp medium for 3 days, and then transferred to -Leu/-Trp/-His/-Ade (with and without X-α-gal) medium for 3 days. Yeast colonies were grown and turned blue on SD/-Leu-Trp-His-Ade containing X-α-gal (0.2 mg/ml) if an interaction existed between the proteins.

### Pull-down assay

The full-length coding region was amplified with appropriate modifications, generating GST-BcNAC153 or HIS-BcCRK1. The constructed recombinant plasmid was transformed into high protein expression *Escherichia coli.* BL21, and then induced protein expression with IPTG. The non-specifically bound proteins are washed away with a buffer that contains detergents and salts to reduce non-specific binding. The obtained fusion protein was affinity chromatographed with glutathione agarose beads, and the purified eluate was detected with SDS PAGE.

### Co-immunoprecipitation assay

*BcNAC153* was inserted into pCAMBIA1302-GFP, and *BcCRK1* was inserted into pCAMBIA1302-MYC. All constructs were transformed into *A. tumefaciens* strain GV3101. The *N. benthamiana* plants with 4–5 young leaves grown at 23°C under LD conditions were used as materials. The total protein was extracted from tobacco expressing BcNAC153-GFP and BcCRK1-MYC using anti-MYC affinity gel, followed by western blotting with anti-GFP, and anti-MYC (Roche).

### Bimolecular fluorescence complementation (BiFC) assay

The coding regions of BcNAC153 and BcCRK1 were amplified and cloned into pSPYNE173 and pSPYCE (M) vectors, respectively. According to the manufacturer’s instructions, the constructs were transferred and made instant expressions into *N. benthamiana* leaves after with injection of *A. tumefaciens* strain GV3101. YFP fluorescence was then detected using a confocal laser scanning microscope (LSM800, Zeiss).

### Subcellular localization

The full-length coding region (minus the termination codon) was amplified with appropriate modifications, generating 35S::GFP-BcNAC153 or 35S::GFP-BcCRK1. It was then transformed into *N. benthamiana* leaves after incubation for 24 h at 28°C. GFP fluorescence was then detected using a confocal laser scanning microscope (LSM800, Zeiss). Control samples were transformed with a pCAMBIA-1302 empty vector.

### Validation of ubiquitination degradation

The ubiquitination and degradation of BcNAC153 by BcCRK1 was tested with proteasome inhibitor MG132 (Selleck, S2619), which can prevent degradation by the 26S proteasome complex. MG132 (10 mM MgCl2, 50 μM MG132) and its reference solution were injected 8 h before collection. A CCD camera was used to observe luciferase activity in *N. benthamiana* leaves.

### Generation of transgenic lines

The subject sequence was introduced into relevant vector by homologous recombination system (ClonExpress^R^Ⅱ One Step Cloning Kit, Vazyme) or restriction endonuclease reaction. Since the *BcNAC153* and *BcCRK1* overexpression line may cause abnormal growth phenotype, so it was constructed into the inducible vector *pMDC30* containing the heat shock promoter element *pHSP*. The pHSP::*BcNAC153* and pHSP::*BcCRK1* transgenes were introduced into Arabidopsis by the *A. tumefaciens* strain EHA105. 1/2MS medium, which contained 50 μg mL^−1^ hygromycin or 1 μg mL^−1^ kanamycin, was applied for transformed progeny selection. Each of three independent T3 transgenic plants was obtained and validated by using the PCR primer pair in Table S7 for amplification.Transient overexpression assays were performed in NHCC001 leaves using a method adapted from previous studies (Wang et al. 2023).

## Supplementary Information

Supplementary Material 1: Fig. S1. F_2_ generation population and Fv/Fm determination for QTL mapping. (a) Phenotype of F_2_ generation population with high temperature treatment (43°C, 6H). (b) A partial display of Fv/Fm value. (c) Frequency distribution of Fv/Fm value for individual plants within the F_2_ population. Fig. S2 Phenotype of *p35S::BcNAC153*transgenic plants before and after 43°C treatment, as well as recovery for 3 days on normal conditions. Fig. S3 *BcNAC153*transient expression negatively regulates high temperature tolerance in *Brassica rapa.* (a) The leaf disc phenotypes of NHCC were observed under normal condition (22°C) and high temperature stress (43°C). (b) Relative expression levels of *BcNAC153 *in transient transferred lines. Values are presented as the means ± SD of three replicates (Student’s t-test, ** *p* < 0.01); the *35S::BcNAC153* compared with the control (*35S::GFP*). (c) Electrolyte leakage of *BcNAC153 *in transient transgenic plants before and after 43°C treatment. Data was represent the mean ± SEM of biological triplicates. Different letters represent a significant difference at *P* < 0.05 (one-way ANOVA with Fisher’s post hoc test). Fig. S4. The promoter analysis of the upstream regulatory region of BcNAC153 with 8 non-heading Chinese cabbage inbred lines. Fig. S5. Amino acid sequence and modification site analysis of BcNAC153. The purple letter “K” (232aa) is the predicted ubiquitination site, and the green letter “T” (239aa) is the predicted phosphorylation site.1aa-171aa, 172aa-231aa, 232aa-246aa, 247aa-344aa, these protein fragments were used to perform segmentation for yeast two-hybrid interaction validation in Fig. 3b.

## Data Availability

The data supporting the findings of this study are available within the paper and its supplementary information files.

## Abbreviations

*NHCC*: Non-heading Chinese cabbage
*QTL*: Quantitative trait locus
*MAS*: Marker-assisted selection
*INAs*: Immune associated nucleus binding protein
*NAC TFs*: NAM/ATAF/CUC transcription factors
*JUB*: JUNGBRUNNEN
*CDPKs/CRKs*: Ca^2+^-dependent protein kinases/CDPK-related kinases
CCaMK: Calcium/calmodulin-dependent protein kinase
*CBLs*: Calcineurin B-like proteins
*HTS*: High temperature stress
*PVE*: Phenotype variation explained
*PCD*: Programmed cell death
*ROS*: Reactive oxygen species
*HSFA1a*: Heat shock transcription factor A-1a​
*FLS*: Flavonol synthase
*NYC*: Non-yellow coloring
*PAO*: Pheophorbide a oxygenase
*SGR*: Stay-green
*CEP*: Cysteine endopeptidase
*BFN*: Bifunctional nuclease
*RbohD*: Respiratory burst oxidase homolog D
*CAT*: Catalase
*βVPE*: β Vacuolar processing enzyme
