# Supplementary material for: Fine-mapping reveals BcNAC153 act as a negative regulator controlling high temperature tolerance in Brassica rapa
Source: Mol Hortic. 2026 Apr 2;6:25. doi: 10.1186/s43897-025-00208-5 (PMC13045127; doi:10.1186/s43897-025-00208-5)
Supplement: Supplementary file 1 — Supplementary Material 1: Fig. S1. F2 generation population and Fv/Fm determination for QTL mapping. (a) Phenotype of F2 generation population with high temperature treatment (43°C, 6H). (b) A partial display of Fv/Fm value. (c) Frequency distribution of Fv/Fm value for individual plants within the F2 population. Fig. S2 Phenotype of p35S::BcNAC153transgenic plants before and after 43°C treatment, as well as recovery for 3 days on normal conditions. Fig. S3 BcNAC153transient expression negatively regulates high temperature tolerance in Brassica rapa. (a) The leaf disc phenotypes of NHCC were observed under normal condition (22°C) and high temperature stress (43°C). (b) Relative expression levels of BcNAC153 in transient transferred lines. Values are presented as the means ± SD of three replicates (Student’s t-test, ** p < 0.01); the 35S::BcNAC153 compared with the control (35S::GFP). (c) Electrolyte leakage of BcNAC153 in transient transgenic plants before and after 43°C treatment. Data was represent the mean ± SEM of biological triplicates. Different letters represent a significant difference at P < 0.05 (one-way ANOVA with Fisher’s post hoc test). Fig. S4. The promoter analysis of the upstream regulatory region of BcNAC153 with 8 non-heading Chinese cabbage inbred lines. Fig. S5. Amino acid sequence and modification site analysis of BcNAC153. The purple letter “K” (232aa) is the predicted ubiquitination site, and the green letter “T” (239aa) is the predicted phosphorylation site.1aa-171aa, 172aa-231aa, 232aa-246aa, 247aa-344aa, these protein fragments were used to perform segmentation for yeast two-hybrid interaction validation in Fig. 3b. [file 43897_2025_208_MOESM1_ESM.docx]

**Supplementary figures**

**
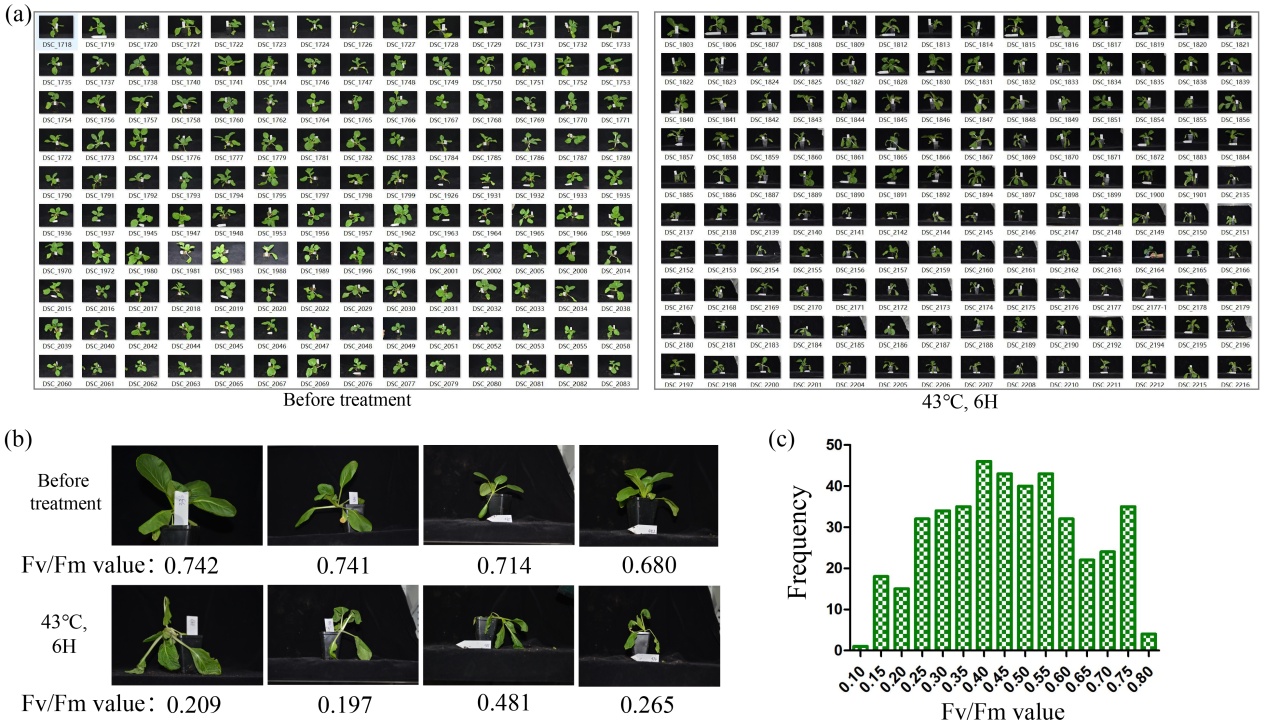
**

**Fig. S1 F_2_ generation population and Fv/Fm determination for QTL mapping.**

(a) Phenotype of F_2_ generation population with high temperature treatment (43°C, 6H). (b) A partial display of Fv/Fm value. (c) Frequency distribution of Fv/Fm value for individual plants within the F_2_ population.

**
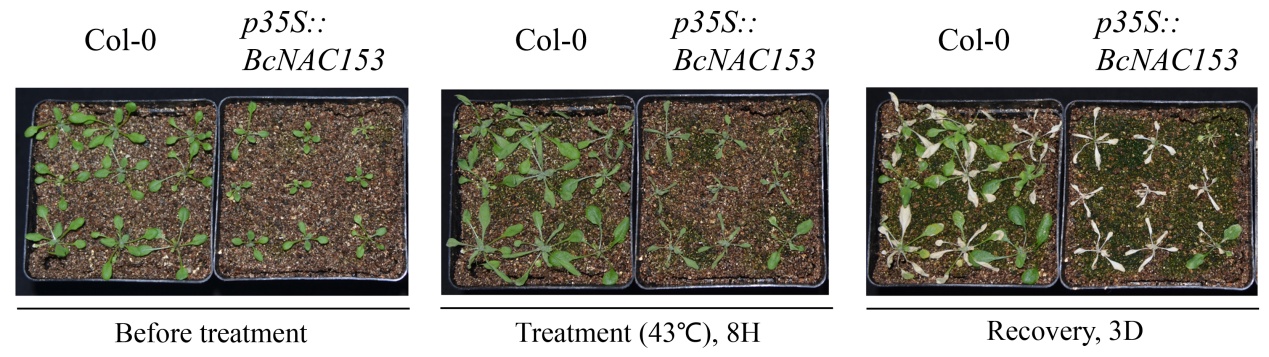
**

**Fig. S2 Phenotype of *p35S::BcNAC153* transgenic plants before and after 43°C treatment，as well as recovery for 3 days on normal conditions.**


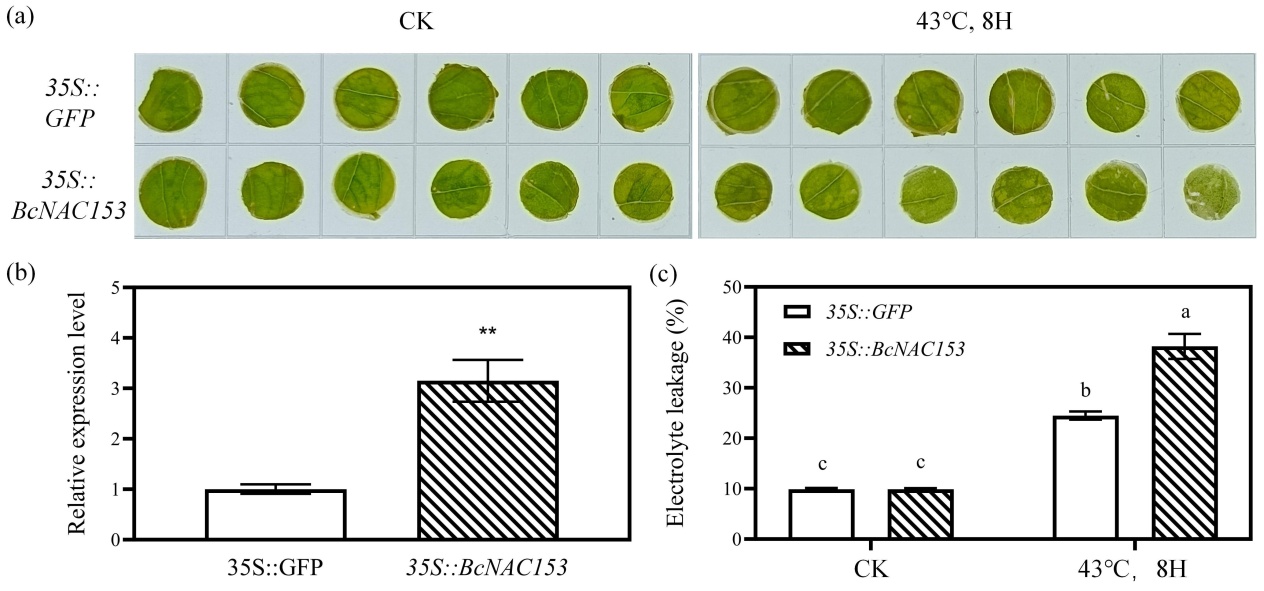


**Fig. S3 *BcNAC153* transient expression negatively regulates high temperature tolerance in *Brassica rapa.*** (a) The leaf disc phenotypes of NHCC were observed under normal condition (22℃) and high temperature stress (43℃). (b) Relative expression levels of *BcNAC153* in transient transferred lines. Values are presented as the means±SD of three replicates (Student’s t-test, ** p < 0.01); the *35S::BcNAC153* compared with the control (*35S::GFP*). (c) Electrolyte leakage of *BcNAC153* in transient transgenic plants before and after 43 ℃ treatment. Data was represent the mean±SEM of biological triplicates. Different letters represent a significant difference at P < 0.05 (one-way ANOVA with Fisher’s post hoc test).

**
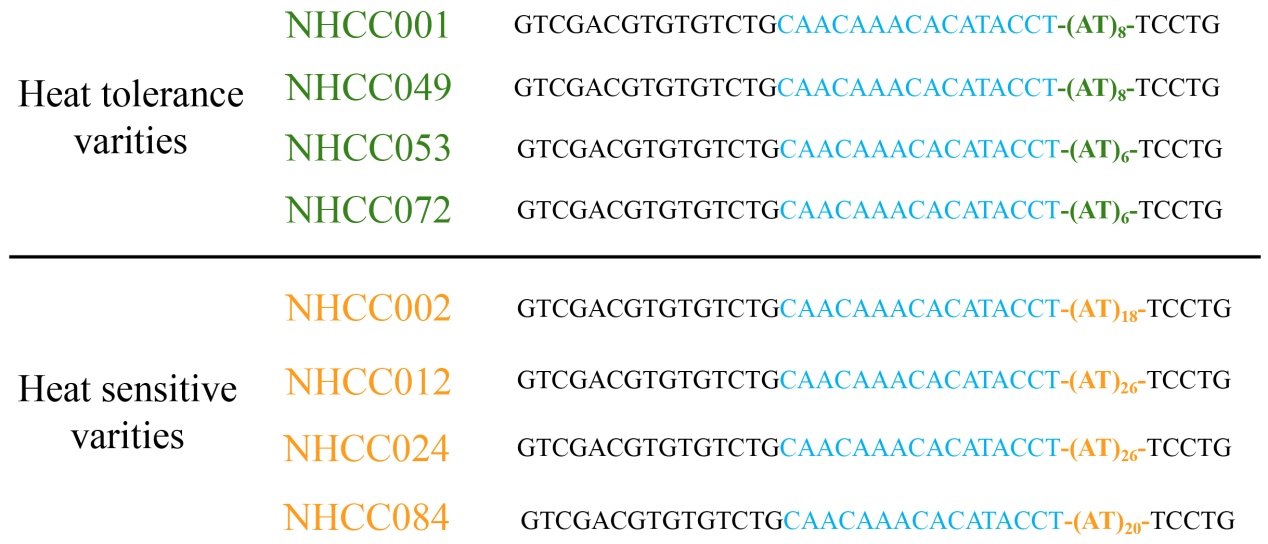
Fig. S4 The promoter analysis of the upstream regulatory region of BcNAC153 with 8 non-heading Chinese cabbage inbred lines.**

**
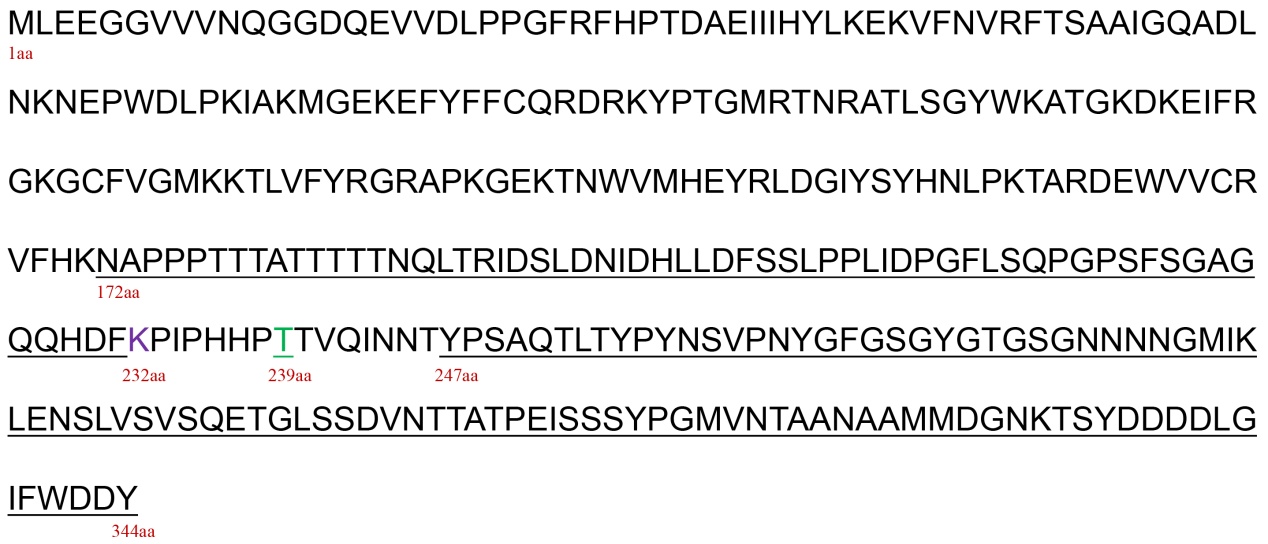
**

**Fig. S5 Amino acid sequence and modification site analysis of BcNAC153.** The purple letter “K” (232aa) is the predicted ubiquitination site, and the green letter “T” (239aa) is the predicted phosphorylation site.1aa-171aa, 172aa-231aa, 232aa-246aa, 247aa-344aa, these protein fragments were used to perform segmentation for yeast two-hybrid interaction validation in Fig. 3b.
